# Pregnenolone, a cholesterol metabolite, induces glioma cell apoptosis via activating extrinsic and intrinsic apoptotic pathways

**DOI:** 10.3892/ol.2014.2147

**Published:** 2014-05-16

**Authors:** XIAO XIAO, LIJUN CHEN, YING OUYANG, WENBO ZHU, PENGXIN QIU, XINWEN SU, YUNLING DOU, LIPENG TANG, MIN YAN, HAIPENG ZHANG, XIAOXIAO YANG, DONG XU, GUANGMEI YAN

**Affiliations:** 1 Department of Pharmacology, Zhongshan School of Medicine, Sun Yat-sen University, Guangzhou, Guangdong 510080, P.R. China; 2 Department of Neonatology, Sun Yat-sen Memorial Hospital, Sun Yat-sen University, Guangzhou, Guangdong 510120, P.R. China; 3 Department of Anesthesiology, The First Affiliated Hospital of Sun Yat-sen University, Guangzhou, Guangdong 510080, P.R. China

**Keywords:** pregnenolone, glioma, apoptosis

## Abstract

Gliomas are one of the most common types of malignant tumors worldwide, however, an effective therapeutic strategy not yet been fully determined. The present study aimed to investigate the anti-glioma activity and underlying mechanisms of pregnenolone, which originates from cholesterol and is metabolized into important steroid hormones in the body. The results demonstrated that 100 μM pregnenolone induced a significant loss of cell viability in various malignant glioma cell lines. In the U-87 MG, LN-18 and C6 cell lines, the loss of cell viability resulted from cell apoptosis, which was evidenced by apoptotic nuclear morphology changes and caspase 3 activation. Moreover, the increased activities of caspase 8 and 9 strongly indicated that pregnenolone activated the extrinsic and intrinsic pathways of apoptosis. Additionally, glioma cell apoptosis was prevented by the general caspase inhibitor, Z-VAD-FMK. In the C6 cells, upregulation of Fas and Fas ligand triggered the activation of the extrinsic pathway, whereas knockdown of Fas significantly abrogated the cell apoptosis that was induced by pregnenolone. Furthermore, downregulation of the anti-apoptotic protein, B-cell lymphoma 2 and upregulation of pro-apoptotic proteins, such as Bax and Bak, activated the intrinsic pathway. In conclusion, pregnenolone induced glioma cell apoptosis in a caspase-dependent manner, which was mediated by activation of the extrinsic and intrinsic apoptotic pathways.

## Introduction

Gliomas originate from glial cells and are the most common type of primary brain tumors accounting for 80% of all malignant primary brain tumors (1). According to the pathological and clinical criteria established by the World Health Organization, gliomas are classified as grades I–IV (2,3). Grade IV tumors, such as glioblastomas (GBMs), are the most devastating and aggressive form comprising >50% of gliomas, and have a poor prognosis (4). The current treatment standard of GBMs is surgical resection to a feasible extent, followed by radiotherapy and chemotherapy. Among the currently available chemotherapy agents, temozolomide is the most popular; doctors and patients favor it as it is administrated orally and efficiently crosses the blood-brain barrier (BBB) (5). However, 67.2–76% of patients are resistant to this agent and therefore do not benefit from it (6). Regardless of systemic therapeutic strategies, including surgery, temozolomide and radiotherapy, patient median survival is only 14.6 months and the five-year survival rate is ~9.8% (7,8). The poor prognosis of glioma fuels the requirement for identifying therapeutic agents with the merits of temozolomide, such as high lipophilicity and strong anti-glioma activity.

**Figure 1:**
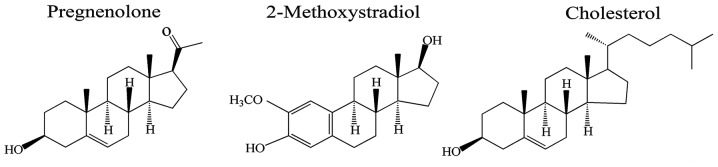
Chemical structure of pregnenolone and 2-methoxyestradiol, the metabolites of cholesterol.

Steroid hormones are generally divided into the following five groups: Estrogens, androgens, progestogens, glucocorticoids and mineralocorticoids (9,10). The natural steroid hormones are predominantly synthesized from cholesterol in the gonads and adrenal glands, and readily diffuse through the cell membrane due to their lipophilic properties (11,12). Accumulating evidence has indicated that certain steroid hormones possess antitumor activities, such as the 17β-estradiol metabolite, 2-methoxyestradiol (2ME), which exerts the strongest activity. 2ME inhibits proliferation and induces apoptosis of various types of cancer, including gliomas, and breast and gastric cancer, independently of estrogen receptors α and β (13–15). However, findings from a clinical trial identified low oral bioavailability of 2ME, which prevents the transfer of this promising agent from bench to bed side (16). The example of 2ME implies the application potential of steroid hormones and provides the basis for the subsequent investigation of other endogenous steroid hormones, such as pregnenolone (Fig. 1).

The present study aimed to investigate the pharmacological effects of the endogenous steroid, pregnenolone, on GBM cells, and the mechanisms underlying its pro-apoptotic activity via the extrinsic and intrinsic apoptotic pathways. Pregnenolone may be a leading compound in the treatment of gliomas and may be modified and developed for clinical application.

## Materials and methods

### Antibodies and reagents

Antibodies against tubulin, B-cell lymphoma 2 (Bcl-2) and Fas ligand (L) were obtained from Cell Signaling Technology, Inc. (Beverly, MA, USA). Bak and Bax antibodies were purchased from Santa Cruz Biotechnology, Inc. (Santa Cruz, CA, USA) and cluster of differentiation (CD)95/Fas antibody was purchased from Epitomics, Inc. (Burlingame, CA, USA). Pregnenolone, Hoechst 33342 and methyl-thiazolyl-tetrazolium (MTT) were purchased from Sigma-Aldrich (St. Louis, MO, USA). The general caspase inhibitor, Z-VAD-FMK, was obtained from EMD Millipore Corp. (Billerica, MA, USA). The Caspase-Glo^®^ 8, 9 and 3/7 Activity assay kits were purchased from Promega Corp. (Madison, WI, USA).

### Cell culture and drug treatment

The C6 rat glioma and U-87 MG and LN-18 human glioma cell lines were obtained from the American Type Culture Collection (Manassas, VA, USA). The cells were cultured in Dulbecco’s modified Eagle’s medium (DMEM; Gibco, Grand Island, NY, USA) supplemented with fetal bovine serum (FBS; Invitrogen Life Technologies, Carlsbad, CA, USA), 100X MEM Non-Essential Amino Acid, GlutaMax™ (Gibco), penicillin (Gibco) and streptomycin (Gibco) in a humidified atmosphere of 5% CO_2_ at 37°C. Pregnenolone was dissolved in ethanol to obtain a 10-mM stock solution and stored at −20°C. For the drug treatment, pregnenolone was diluted in DMEM and added at different concentrations. Ethanol at corresponding concentrations served as the vehicle control.

### Cell viability and cytotoxicity assays

Cell viability was determined via MTT assay. The cells that were growing in logarithmic phase were seeded in 96-well plates and treated with pregnenolone. MTT (10 μl; 0.5 mg/ml) was added to each well, which was subsequently incubated at 37°C for 4 h to allow the yellow dye to transform into blue crystals. The medium was removed and 100 μl dimethyl sulfoxide (DMSO; Sigma-Aldrich) was added to each well to dissolve the dark blue crystals. The optical density was measured with a microplate reader (iMark™, Bio-Rad, Hercules, CA, USA) at 490 nm. Five replicates were prepared for each condition.

Lactate dehydrogenase (LDH) release was quantified with a CytoTox 96^®^ Non-Radioactive Cytotoxicity assay kit (Promega Corp.) according to the manufacturer’s instructions. Plates were incubated with an LDH substrate at room temperature for 30 min in the dark and absorbance was measured at 490 nm with a microplate reader (iMark™, Bio-Rad).

### Caspase 3/7, 8 and 9 activities assay

The glioma cells were seeded into 96-well plates at 3,000–5,000 cells/well and incubated for 24 h. The cells were treated with pregnenolone at different concentrations. After 24 h of treatment, the enzymatic activity of caspase 8, 9 and 3/7 was determined using the Caspase-Glo^®^ 8 and 9 Activity assay kit and the Caspase-Glo^®^ 3/7 Activity assay kit, respectively, according to the manufacturer’s instructions. Simultaneously, the cells were plated in 96-well plates and treated with pregnenolone for 24 h. The relative cell number was measured by MTT assay and evaluated as follows: Caspase activities = total enzymatic activity values of caspase/cell number.

### Hoechst 33342 staining and terminal deoxynucleotidyl transferase dUTP nick end labeling (TUNEL) assay

The glioma cells were stained with Hoechst 33342 (5 μg/ml) at 37°C for 20 min in the dark and photographed under a fluorescent microscope (IX71, Olympus Corp., USA) with a 340-nm excitation filter. Apoptotic cells were characterized as demonstrating condensed nuclei and cell shrinkage. The proportion of apoptotic cells in total cells was quantified in three randomly selected microscopic fields.

For the TUNEL assay (Roche Diagnostics GmbH, Mannheim, Germany), the glioma cells were fixed in 4% paraformaldehyde solution at room temperature for 1 h. The cell samples were detected via an *In Situ* Cell Death Detection kit, TMR red (Roche Diagnostics GmbH) according to the manufacturers’ instructions and analyzed under a fluorescent microscope (Olympus Corp.) with a 540-nm excitation filter. A red fluorescence signal was observed in the apoptotic cells.

### Immunoblotting

Immunoblotting was performed as previously described (17). Briefly, following cell lysis and measurement of protein concentrations, the cells were dissolved in sodium dodecyl sulfate (SDS) sample buffer (Beyotime Institute of Biotechnology, Shanghai, China). Equal amounts of protein were analyzed by SDS-PAGE on 12% polyacrylamide gels (Bio-Rad) and the proteins were electroblotted onto polyvinylidene fluoride membranes (Roche Diagnostics GmbH). The membranes were incubated in 5% non-fat dry milk in Tris-buffered saline (NaCl/Tris; Boster Biological Engineering Co., Ltd., Wuhan, China) containing 0.1% Tween-20 (Sigma-Aldrich) for 1 h at room temperature overnight at 4°C, and subsequently incubated with primary antibodies. Following incubation with a horseradish peroxidase-labeled secondary antibody, protein exposure was achieved with a molecular imager (ChemiDOC™ XRS+, Bio-Rad).

### Small interfering (si)RNA-mediated knockdown of Fas expression

Fas-siRNA (si-Fas) and negative control (NC) oligonucleotides were purchased from Guangzhou RiboBio Co., Ltd. (Guangzhou, China). The C6 cells of 30–50% confluence were transfected by Lipofectamine^®^ RNAiMAX (Invitrogen Life Technologies) according to the manufacturer’s instructions and siRNA inhibitory efficacy was examined by immunoblotting.

### Statistical analysis

Data are expressed as means ± standard deviation of the three separate experiments. One-way analysis of variance and Student’s t-test were used to determine the differences between the the two groups. P<0.05 was considered to indicate a statistically significant difference.

## Results

### Pregnenolone decreases cell viability of malignant glioma cells

**Figure 2:**
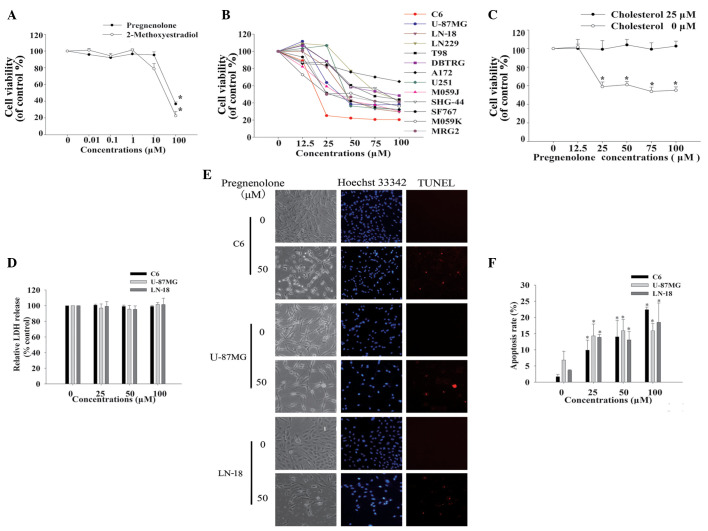
Pregnenolone decreased cell viability in glioma cells by inducing apoptosis. (A) Glioma cells were treated with different doses of pregnenolone for 48 h. Cell viability was determined by the MTT assay. Similar to 2ME, pregnenolone decreased cell viability at 100 μM in the U-87 MG human glioma cell line (n=3; ^*^P<0.001, Bonferroni t-test). (B) Pregnenolone decreased cell viability at 48 h in a series of glioma cell lines. (C) Exogenous cholesterol (25 μM) prevented the pregnenolone-induced cell loss in U-87 MG cells at 48 h (^*^P<0.05). (D) The C6, LN-18 and U-87 MG glioma cells were treated with different concentrations of pregnenolone for 48 h (n=3). No significant LDH release changes were observed in the cytotoxicity assay. (E) Pregnenolone induced glioma cell apoptosis after 48 h showing characteristics of nuclear condensation and DNA breakage. (F) Bar chart of the apoptosis rate (%) in glioma cells at 48 h (^*^P<0.05). 2ME, 2-methoxyestradiol; LDH, lactate dehydrogenase; TUNEL, terminal deoxynucleotidyl transferase dUTP nick end labeling. ^*^P<0.001 vs 0 μM pregnenolone.

First, the effect of pregnenolone on the growth of U-87 MG human GBM cells was investigated. The cell numbers were greatly reduced following treatment with 100 μM pregnenolone for 48 h (Fig. 2A) and the growth inhibitory efficacy was comparable with 2-ME. To further investigate the dose-dependent effect of pregnenolone on the anti-glioma spectrum, a series of glioma cell lines, including C6, LN-18 and T98, were treated with pregnenolone at different doses (0, 12.5, 25, 50, 75 and 100 μM) for 48 h. Notably, pregnenolone dose-dependently decreased the cell viability in all the glioma cell lines at varying degrees (Fig. 2B). The dose-dependent decline of cell viability in the majority of the cell lines was more sensitive between 12.5 and 50 μM pregnenolone compared with between 50 and 100 μM pregnenolone. Therefore, the glioma cells were treated with doses between 12.5 and 50 μM pregnenolone to further investigate the detailed mechanisms of action. Exogenous cholesterol may completely prevent the pregnenolone-induced cell loss in U-87MG cells (Fig. 2C).

### Pregnenolone induces glioma cell apoptosis

To investigate the mechanisms underlying the loss of cell viability induced by pregnenolone, further experiments in the C6, U-87 MG and LN-18 glioma cell lines were conducted. The glioma cells were incubated with pregnenolone at different concentrations for 48 h, and examined via cytotoxicity assay, Hoechst 33342 staining and TUNEL assay. No significant changes in LDH release were observed in the pregnenolone-treated glioma cells, indicating that pregnenolone did not result in cell necrosis (Fig. 2D). Compared with the control group, the condensed chromatin in the nucleus of the 50 μM pregnenolone-treated group appeared smaller and brighter under the fluorescent microscope (Fig. 2E). The apoptosis rate in the pregnenolone-treated groups increased in a dose-dependent manner (Fig. 2F). Following the TUNEL assay, there were more positive signals observed in the cells that were treated with pregnenolone (Fig. 2E). These findings support the conclusion that pregnenolone induced glioma cell apoptosis in a dose-dependent manner.

### Pregnenolone leads to caspase-dependent apoptosis via extrinsic and intrinsic apoptotic pathways

**Figure 3:**
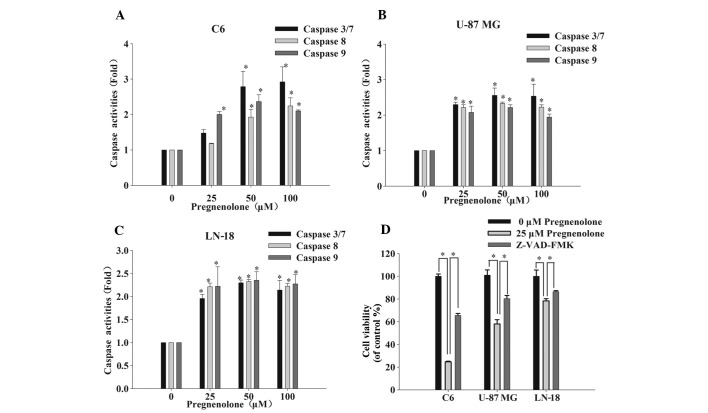
Pregnenolone induced apoptosis by the activation of caspases. The C6, LN18 and U-87MG glioma cells were incubated with different concentrations (0, 25, 50 and 100 μM) of pregnenolone for 48 h (n=3). The activation of caspase 3/7, 8 and 9 were observed in (A) C6, (B) U-87 MG and (C) LN-18 (^*^P<0.05). (D) General caspase inhibitor, Z-VAD-FMK, at 50 μM partly prevented glioma cell apoptosis induced by pregnenolone. ^*^P<0.05 vs. 0μM pregnenolone group..

Previous studies have reported that anticancer therapies eventually result in the activation of caspases, a family of cysteine proteases that act as common death effector molecules in the apoptotic pathway (18–20). To determine whether the apoptotic pathway is induced by pregnenolone, the activity of caspase 8 and 9, and 3/7 in the death receptor (extrinsic) and mitochondria-dependent (intrinsic) pathways, respectively, was investigated. Caspase 9 is an important intracellular amplifier of caspase signaling that is downstream of mitochondria and caspase 8 is an apical caspase in death receptor signaling, which has been well-established (21). After 24 h of exposure to pregnenolone, the activity of caspase 8 and 9 markedly increased compared with the control group. The activity of the downstream effector caspase 3/7 increased 2–3-fold following a 48-h exposure to pregnenolone (Fig. 3A–C). To determine whether apoptosis was due to the activation of caspases, the general caspase inhibitor, Z-VAD-FMK, was applied to the pregnenolone-treated glioma cells and cell viability was measured via an MTT assay. As expected, 50 μM Z-VAD-FMK prevented the loss of cell viability that was caused by pregnenolone (Fig. 3D).

### Pregnenolone induces apoptosis by decreasing Bcl-2 and increasing Fas/FasL activity

**Figure 4:**
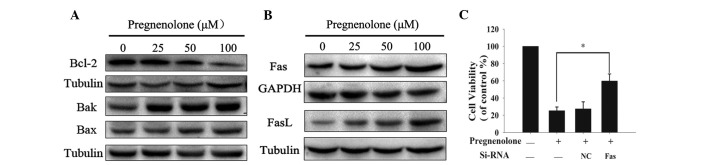
Pregnenolone-induced apoptosis was mediated by regulating Fas, FasL and Bcl-2 family members. Western blot analysis was performed in C6 cells after 48 h of incubation with pregnenolone at different concentrations (n=3). (A) Pregnenolone decreased the protein expression of Bcl-2 and increased the levels of Bak and Bax. (B) Upregulation of Fas and FasL was observed in the pregnenolone-treated groups. (C) Knockdown of Fas partly rescued the cell viability-loss by pregnenolone. ^*^P<0.05 vs. pregnenolone-treated group without siRNA. Bcl-2, B-cell lymphoma 2; FasL, fas ligand; siRNA, small interfering RNA.

The present study demonstrated that pregnenolone triggers the intrinsic and extrinsic apoptotic pathways by activating caspase 8 and 9. To investigate the mechanisms underlying pregnenolone-induced cell apoptosis, the upstream targets in the mitochondria- and death receptor-mediated pathways were analyzed. First, the protein levels of the Bcl-2 family members, which are responsible for the stability of the mitochondrial membrane, were examined. The data demonstrated that the anti-apoptotic protein, Bcl-2, was markedly downregulated, while the pro-apoptotic factors, Bax and Bak, were upregulated in the pregnenolone-treated C6 cells (Fig. 4A). In addition, pregnenolone treatment resulted in a marked increment of the activity of Fas and FasL, an important death receptor and ligand, respectively that are involved in the extrinsic pathway (Fig. 4B). To confirm whether pregnenolone-induced apoptosis was mediated by Fas and FasL, Fas was silenced in the C6 cells, using siRNA, and the cell viability was measured (Fig. 4D). Compared with the cell viability loss that was observed in the control group, Fas knockdown significantly decreased the cell apoptosis, which was induced by pregnenolone. These findings indicated that pregnenolone may trigger the intrinsic and extrinsic apoptotic pathways by regulating the Bcl-2 family, Fas and FasL.

## Discussion

Pregnenolone, a neurosteroid, is synthesized in the nervous system (22,23), arises from cholesterol and is metabolized into important steroid hormones (24). Due to its chemical structure, pregnenolone is highly lipid-soluble and rapidly crosses the BBB (25). The concentration of pregnenolone in overall brain tissue is 74-fold higher compared with that in plasma (26). Notably, the findings of the present study indicated that this endogenous steroid hormone is able to induce glioma cell apoptosis. The antitumor efficacy of pregnenolone is as strong as 2-ME, however, whether it may be developed into an anti-glioma agent requires further investigation, as does its bioavailability.

In addition to pregnenolone and 2-ME, previous studies have reported that a number of other steroid hormones originating from cholesterol exhibit antineoplastic properties, such as dehydroepiandrosterone (DHEA) (27) and cholesteryl sulfate (28), the underlying mechanisms of which are distinct. Furthermore, DHEA induced apoptotic and necrotic death in cervical cancer cells (29). In the present study, pregnenolone induced glioma cell apoptosis, but not necrotic death. Additionally, 2-ME upregulated death receptor 5 and induced apoptosis through activation of the extrinsic apoptotic pathway (13). However, the present study demonstrated that pregnenolone upregulated Fas and FasL, and induced apoptosis through the activation of the extrinsic pathway. Moreover, the intrinsic apoptotic pathway was activated by pregnenolone, which was characterized by downregulation of anti-apoptotic Bcl-2 expression, upregulation of pro-apoptotic Bax and Bak expression, and activation of caspase 9.

Notably, cholesterol blocked the loss of cell viability that was induced by pregnenolone (Fig. 2C), demonstrating the critical role of cholesterol in apoptosis. As a neutral lipid, cholesterol is indispensable in the regulation of cell membrane properties in mammalian cells, contributes to the unique biophysical properties of the lipid raft microdomain and is mechanistically important for signal transduction by raft proteins (30). Lipid rafts, incorporating distinct classes of proteins, are comprised of cholesterol and sphingolipids in the exoplasmic leaflets of the bilayer (31,32). Disruption of lipid rafts by dispersion or extraction of membrane cholesterol results in inhibition of raft-dependent signaling events and eventually affects cell survival. It was reported that simvastatin, a cholesterol synthesis inhibitor, lowered raft cholesterol content and induced apoptosis in prostate cancer cells; however, replenishing cell membranes with cholesterol reversed these inhibitory and pro-apoptotic effects (33). In addition, previous studies have shown that ginsenoside Rh2, containing a cholesterol backbone with hydroxyl groups, induced ligand-independent Fas activation and activated caspase 8 via lipid raft disruption (34,35). Similar to Rh2, other cholesterol derivatives, such as desmosterol treatment, resulted in the disappearance of caveolae and blocked insulin receptor activation via lipid raft disruption (36). As a cholesterol metabolite, pregnenolone has a similar chemical structure to cholesterol, and may interrupt normal lipid rafts and trigger apoptosis.

The Fas/FasL and mitogen-activated protein kinase (MAPK) signaling pathways are two key cell death and survival signaling pathways, which are influenced by the alteration of lipid rafts (37). In the present study, Fas/FasL signaling was dramatically potentiated (Fig. 4), while EGFR/Ras/MAPK signaling remained stable (data not shown). Moreover, an elevation in Fas/FasL signaling was confirmed as a critical cause for pregnenolone-induced apoptosis by si-Fas. The mechanisms by which pregnenolone upregulates Fas and FasL, and whether pregnenolone disrupts lipid rafts require further investigation.

In conclusion, the present study demonstrated that pregnenolone, an endogenous steroid hormone, exerts a strong anti-glioma effect by inducing apoptosis. Pregnenolone treatment resulted in caspase-dependent apoptosis via the extrinsic and intrinsic apoptotic pathways in glioma cells. Based on this anti-glioma activity and high BBB permeability, pregnenolone may be a promising compound for, and aid in the development of, novel agents for anti-glioma therapy.
